# Male reproductive hormones in patients treated with pretomanid

**DOI:** 10.5588/ijtld.21.0654

**Published:** 2022-06-01

**Authors:** K. Boekelheide, M. Olugbosi, J. Nedelman, D. Everitt, E. Smith, M. Betteridge, E. Sun, M. Spigelman

**Affiliations:** 1 Brown University, Providence, Rhode Island, USA; 2 TB Alliance, Pretoria, South Africa; 3 TB Alliance, New York, NY; 4 RTI International, Research Triangle Park, NC, USA

**Keywords:** pretomanid, male hormones

## Abstract

**BACKGROUND:**

Pretomanid (Pa) is a nitroimidazole-class drug recently approved by the US Food and Drug Administration and other regulatory authorities as part of a regimen for treating highly drug-resistant pulmonary *Mycobacterium tuberculosis* infections. Studies in rodents identified the testis as a target organ of concern, which led to monitoring of reproductive hormones in >800 male patients enrolled in four clinical trials of Pa-containing regimens and the HRZE (isoniazid+rifampin+pyrazinamide+ethambutol) control regimen.

**METHODS:**

Serum hormone levels relevant to male reproductive health – follicle stimulating hormone (FSH), luteinizing hormone (LH), inhibin B (InhB) and total testosterone (T) – from the four clinical trials were summarized numerically and analyzed by repeated-measures modeling.

**RESULTS:**

Hormone levels generally behaved similarly in Pa-containing and HRZE arms. Relative to baseline, serum T and InhB levels generally increased at the end of treatment and follow-up. FSH and LH levels were variable, but were generally at or below baseline levels by follow-up. Before treatment, many patients were borderline hypogonadal, with T levels near the lower limits of the normal range.

**CONCLUSION:**

Changes in male hormones in four clinical trials studying patients with TB indicate that Pa-containing treatment was not associated with testicular toxicity but rather led to improvement in the underlying hypogonadism.

## Abstract (fr)

**CONTEXTE:**

Le prétomanide (Pa) est un médicament de la classe des nitroimidazoles, récemment homologué par la FDA américaine et d’autres autorités réglementaires comme agent d’un schéma thérapeutique pour les infections pulmonaires à *Mycobacterium tuberculosis* hautement résistant aux antituberculeux. Les études réalisées chez les rongeurs ont permis d’identifier les testicules comme organes cibles préoccupants, entraînant la surveillance des hormones de reproduction chez >800 hommes inclus dans quatre essais cliniques visant à évaluer les schémas à base de Pa et le schéma de contrôle à base d’HRZE (isoniazide+rifampicine+pyrazinamide+ethambutol).

**MÉTHODE:**

Les taux sériques d’hormones pertinentes à la santé reproductrice de l’homme (hormone folliculo-stimulante [FSH], hormone lutéinisante (LH), inhibine B [InhB] et testostérone totale [T]) issus des quatre essais cliniques ont été synthétisés numériquement et analysés à l’aide de modélisations à mesures répétées.

**RÉSULTATS:**

Les taux d’hormones étaient généralement similaires entre les groupes sous schéma à base de Pa et sous schéma à base de HRZE. Par rapport à l’inclusion, les taux de T et d’InhB avaient tendance à augmenter à la fin du traitement et pendant le suivi. Les taux de FSH et de LH étaient variables mais, au moment du suivi, ils étaient généralement similaires ou inférieurs aux taux observés à l’inclusion. Avant le traitement, de nombreux patients étaient à la limite de l’hypogonadisme, avec des taux de T proches de la limite inférieure de la normale.

**CONCLUSION:**

Les variations des taux hormonaux observées chez l’homme dans le cadre de quatre essais cliniques évaluant des patients atteints de tuberculose, indiquent que le traitement à base de Pa n’est pas associé à une toxicité testiculaire mais plutôt à une amélioration de l’hypogonadisme sous-jacent.

About 25% of the world’s population is infected with *Mycobacterium tuberculosis*, according to WHO estimates,^1^ and *M. tuberculosis* is a major cause of infectious disease-related deaths worldwide. In the past decade, two new drugs have received FDA approval for the treatment of *M. tuberculosis*: bedaquiline (BDQ), approved in 2012, and pretomanid (Pa), the focus of this study. Pa was approved by US Food and Drug Administration under the Limited Population Pathway for Antibacterial and Antifungal Drugs (LPAD pathway) in 2019 for the treatment of adults with extensively drug-resistant TB or treatment-intolerant or non-responsive multidrug-resistant TB, in combination with BDQ and linezolid.^2^ The combination of the three drugs has markedly improved the outlook for TB therapy in patients with highly drug-resistant (DR) disease.^3^

Patients with TB frequently have a chronic, debilitating illness, and often present with endocrine dysfunction. Male hypogonadotropic hypogonadism is a common condition in patients with TB,^4^,^5^ and may be a consequence of infection of endocrine system organs or of long-standing disease accompanied by weight loss and poor nutrition.^6^ Male patients with malnutrition are recognized to have lower testosterone (T) levels associated with lower body mass index (BMI), consistent with primary hypogonadism, while luteinizing hormone (LH) levels are negatively associated with BMI.^7^,^8^ Thus, patients presenting with TB are likely to have abnormalities in hypothalamic-pituitary-gonadal (HPG) axis function and hormones.

Pa is a member of the nitroimidazole class of antibiotics.^9^ Medicinal chemists have generated hundreds of nitroimidazole congeners, a chemical class which is recognized as sometimes causing male reproductive tract toxicity in preclinical studies in rodents.^10^ An example is metronidazole (MET), which the FDA approved in the 1960s and commonly used to treat susceptible anaerobic infections throughout the world.^11^ MET caused male reproductive tract toxicity in rats through inhibition of spermatogenesis, an effect that was only partially reversible.^12^,^13^ MET also produced male reproductive tract toxicity in mice with inhibited spermatogenesis and altered fertility.^14^ Male reproductive tract effects in preclinical studies have also been observed with other nitroimidazoles.^15^–^17^ The results and interpretation of the male reproductive tract effects in four clinical studies of Pa are presented here.

## METHODS

The Global Alliance for TB Drug Development (TB Alliance; New York, NY, USA) has conducted multiple Phase 1, 2 and 3 clinical studies to characterize Pa’s safety, pharmacokinetics (PK), and anti-TB activity as monotherapy and in combination with other anti-mycobacterial agents. All clinical studies supporting Pa were conducted under Good Clinical Practice; all patients provided written informed consent, and all studies were approved by local and national Ethics Committees *(*see Supplementary Table S8 for full list of Ethics Committees*)*.

Male reproductive hormones were assessed in four clinical studies: NC-002,^18^ NC-005,^19^ NC-006 (Shortening Treatment by Advancing Novel Drugs or STAND),^20^ and NC-008 (SimpliciTB; Clinical-Trials.gov Identifier: NCT03338621) (see Table 1 for details). In these studies, Pa was administered in combination with pyrazinamide (Z, PZA), together with moxifloxacin (PaMfxZ), bedaquiline (BdqPaZ), or both (BdqPaMfxZ). The duration of Pa administration was 4–6 months in NC006 and NC008, and for 2 months in NC002 and NC005. The trials included a standard-of-care comparison group treated with the four-drug regimen of isoniazid (H, INH), rifampin (R, RIF), PZA, and ethambutol (E) (HRZE) given for 2 months (all studies), followed by INH and RIF for 4 months (NC-006 and NC-008). Serum levels of FSH, LH, T (total), and InhB were included in studies NC-006 and NC-008. NC-002 monitored FSH, LH, and T. NC-005 included only FSH. Samples were collected on designated days during each patient’s course of treatment and processed and analyzed using standard clinical protocols.

**Table 1:** Pa clinical program relevant to male reproductive tract

Study name, number, status*	Regimen(s) tested	Title
Phase 3 studies in subjects with MDR-TB or DS-TB		
STAND, NC-006, complete	PaMfxZ Patients enrolled: (*n* = 284) Male patients (*n* = 197; 69% of enrolled population) DS-TB DR-TB Controls: HRZE/HR^†^ DS-TB	A Phase 3 open-label partially randomized trial to evaluate the efficacy, safety and tolerability of the combination of MFX plus PA-824 plus PZA after 4 and 6 months of treatment in adult subjects with drug-susceptible, smear-positive pulmonary TB and after 6 months of treatment in adult subjects with multidrug-resistant, smear-positive pulmonary TB^19^
Phase 2 studies in subjects with DS-TB or MDR-TB		
SimpliciTB, NC-008, ongoing	BdqPaMfxZ Patients enrolled: (*n* = 455) Male patients (*n* = 324; 71% of enrolled population) DS-TB (150 patients) DR-TB (152 patients) Control: HRZE/HR DS-TB (153 patients)	An open-label, partially randomized trial to evaluate the efficacy, safety and tolerability of a 4-month treatment of BDQ plus Pa plus MFX plus PZA (BdqPaMfxZ) compared to a 6-month treatment of HRZE (control) in adult participants with drug-susceptible, smear-positive pulmonary TB (DS-TB) and a 6-month treatment of BdqPaMfxZ in adult participants with drug resistant, smear-positive pulmonary TB (DR-TB) (ClinicalTrials.gov Identifier: NCT03338621)
NC-005/complete	BdqPaZ and control (HRZE/HR) Patients enrolled: (*n* = 240) Male patients (*n* = 182; 76% of enrolled population) DS-TB (180 patients; BdqPaMfxZ) DR-TB (60 patients)	A Phase 2 open-label partially randomized trial to evaluate the efficacy, safety and tolerability of combinations of BDQ, MFX, PA-824 and PZA During 8 weeks of treatment in adult subjects with newly diagnosed drug-susceptible or multidrug-resistant, smear-positive pulmonary TB^18^
NC-002/complete	PaMfxZ Patients enrolled: (*n* = 207) Male patients (*n* = 135; 65% of enrolled population) DS-TB (122 patients) DR-TB (26 patients) Control: HRZE/HR^†^ DS-TB (59 patients)	A Phase 2 open-label partially randomized trial to evaluate the efficacy, safety, and tolerability of the combination of MFX plus PA-824 plus PZA after 8 weeks of treatment in adult patients with newly diagnosed drug-susceptible or multidrug-resistant, smear-positive pulmonary TB^17^

* Not all studies had both a study name and study number.

^†^ Initial 2-month regimen of HRZE followed by a 4-month regimen of HR.

MDR-TB = multidrug-resistant TB; DS-TB = drug-susceptible TB; Pa = pretomanid; MFX = moxifloxacin; Z, PZA = pyrazinamide; DR-TB = drug-resistant TB; H = isoniazid; R = rifampin; E = ethambutol; BDQ = bedaquiline.

The laboratory reference values for hormones in NC-008 were as follows: FSH, 1.5–12.4 IU/L; LH, 1.7–8.6 IU/L; total T, 8.6–29.0 nmol/L; and InhB, <326 pg/mL, and a similar range of reference values applied to the other studies. Intra-individual differences in repeated hormone measurements, such as screening and Day 1 values of hormones in NC-008, were generally small (Supplementary Table S1). Median ages in the four studies were 28–35 years. T levels were also compared to a published reference range for men aged 19–39 years, where the 5^th^,25^th^, 50^th^,75^th^, and 95^th^ percentiles were 9.5, 13.7, 17.6, 21.7, and 28.9 nmol/L, respectively.^21^ BMI values were categorized as underweight and normal depending on whether BMI was below or above 18.5 kg/m^2^.^22^

Hormone levels and BMI were summarized by median values and numbers of subjects at baseline, end of treatment (EOT), and on-treatment and post-treatment follow-up (FU) visits when available (Tables 2–5).

**Table 2:** Summary of serum hormones and BMI in NC-002

		Baseline		Day 57 = EOT	
	
		*n*	Median	*n*	Median
FSH	Pa_100_MfxZ*	28	5.7	25	6.7
	Pa_200_MfxZ^†^	35	5.7	36	4.6
	HRZE^‡^	31	6.1	33	5.6
LH	Pa_100_MfxZ*	28	6.6	25	5.0
	Pa_200_MfxZ^†^	35	5.7	36	4.8
	HRZE^‡^	31	6.8	33	5.0
T	Pa_100_MfxZ*	28	12.0	26	18.5
	Pa_200_MfxZ^†^	35	14.4	36	18.8
	HRZE^‡^	31	14.7	33	32.4
BMI	Pa_100_MfxZ*	28	17.9	26	18.9
	Pa_200_MfxZ^†^	34	18.5	36	20.2
	HRZE^‡^	31	18.1	33	19.0

* Group treated with 100 mg/d Pa (drug-susceptible).

^†^ Group treated with 200 mg/d Pa (drug-susceptible and multidrug-resistant).

^‡^ Group treated with standard of care (drug-susceptible).

BMI = body mass index; EOT = end-of-treatment; FSH = follicle stimulating hormone; Pa or PA-824 = pretomanid; MFX = moxifloxacin; Z = pyrazinamide; H = isoniazid; R = rifampin; E = ethambutol; LH = luteinizing hormone; T = testosterone (total).

**Table 3:** Summary of serum FSH and BMI in NC-005 *

		Baseline		Day 29		Day 57 = EOT		Day 70	
			
		*n*	Median	*n*	Median	*n*	Median	*n*	Median
FSH	DS-TB: Bdq_Load_Pa_200_Z	45	5.0	43	5.7	37	5.6	35	5.8
	DS-TB: Bdq_200_Pa_200_Z	47	5.2	41	6.3	40	5.8	39	6.3
	DR-TB: Bdq_200_Pa_200_MfxZ	36	5.6	37	6.1	38	6.4	37	5.7
	DS-TB: HRZE	46	4.9	45	5.9	43	5.5	43	5.3
BMI	DS-TB: Bdq_Load_Pa_200_Z	43	19.3	43	19.4	37	19.6	35	19.8
	DS-TB: Bdq_200_Pa_200_Z	45	19.2	41	19.7	40	20.0	39	20.2
	DR-TB: Bdq_200_Pa_200_MfxZ	36	18.0	37	18.3	38	18.8	37	18.4
	DS-TB: HRZE	46	17.9	45	18.7	43	18.9	43	19.1

* All patients in the Pa-containing arms (DS-TB and DR-TB) received 200 mg of Pa.

FSH = follicle stimulating hormone; BMI = body mass index; EOT = end-of-treatment; DS-TB = drug-susceptible TB; BDQ = bedaquiline; Pa = pretomanid; Z = pyrazinamide; DR-TB = drug-resistant TB; MFX = moxifloxacin; H = isoniazid; R = rifampin; E = ethambutol.

**Table 4:** Serum hormones and BMI in NC-006

		Baseline		Week 1		Week 2		Week 4		Week 8		Week 12		Week 17		Week 26	
							
		*n*	Median	*n*	Median	*n*	Median	*n*	Median	*n*	Median	*n*	Median	*n*	Median	*n*	Median
FSH	Pa_100_MfxZ, Week 17*	51	5.9	51	6.5	51	7.7	50	6.4	50	6.8	45	7.1	45	7.9	37	5.9
	Pa_200_MfxZ, Week 17^†^	48	6.7	47	8.7	46	8.7	47	9.2	47	8.1	46	7.7	44	7.2	30	7.2
	Pa_200_MfxZ, Week 26^‡^	50	6.4	49	7.6	47	8.5	47	9.2	44	6.9	45	6.8	27	6.6	37	6.3
	HRZE, Week 17^§^	48	5.4	48	7.1	47	6.8	48	6.8	45	6.4	44	6.4	23	5.8	43	5.6
InhB	Pa_100_MfxZ, Week 17*	51	117.7	51	109.9	50	117.4	50	134.6	48	137.9	46	137.3	45	130.0	34	129.2
	Pa_200_MfxZ, Week 17^†^	48	98.7	47	94.0	46	95.7	48	104.2	48	115.9	46	118.9	45	130.4	28	129.2
	Pa_200_MfxZ, Week 26^‡^	50	109.6	48	106.1	47	101.6	47	118.9	43	149.2	47	142.6	27	161.0	38	151.0
	HRZE, Week 17^§^	48	123.0	47	110.8	46	123.9	45	129.8	46	146.1	44	152.9	25	155.4	43	153.2
LH	Pa_100_MfxZ, Week 17*	51	5.7	51	5.5	51	5.0	50	5.1	50	5.4	46	4.6	46	5.7	37	5.0
	Pa_200_MfxZ, Week 17^†^	48	5.8	47	6.0	46	5.6	47	6.2	47	5.5	46	5.0	44	5.2	30	5.4
	Pa_200_MfxZ, Week 26^‡^	50	6.7	49	6.6	47	5.9	47	6.5	43	5.7	46	5.9	27	5.0	37	5.9
	HRZE, Week 17^§^	48	5.6	48	5.3	47	5.8	48	5.7	45	4.6	43	5.6	23	5.2	43	6.0
T	Pa_100_MfxZ, Week 17*	51	9.5	51	13.5	50	13.9	50	13.2	50	15.9	46	14.5	46	15.1	36	17.8
	Pa_200_MfxZ, Week 17^†^	48	9.5	47	12.7	46	12.0	47	11.6	47	12.8	46	13.7	44	15.3	30	17.9
	Pa_200_MfxZ, Week 26^‡^	50	10.6	49	15.0	47	12.0	47	14.8	44	15.7	45	17.0	27	15.3	35	18.7
	HRZE, Week 17^§^	48	11.5	48	20.0	47	21.7	48	22.6	45	25.5	44	26.6	23	23.2	42	24.8
BMI	Pa_100_MfxZ, Week 17*	51	18.6	51	18.8	51	18.8	50	19.3	50	19.2	47	19.5	47	19.5	37	19.5
	Pa_200_MfxZ, Week 17^†^	48	18.0	47	18.2	46	18.2	48	18.7	48	19.0	47	19.4	45	19.5	30	19.2
	Pa_200_MfxZ, Week 26^‡^	50	18.3	49	18.6	47	18.6	46	18.9	44	18.9	48	19.4	27	20.4	38	19.1
	HRZE, Week 17^§^	48	18.9	48	18.7	47	19.0	48	19.3	46	19.6	44	20.6	25	20.9	43	20.4

* Pa 100 mg/d for 17 weeks (DS).

† Pa 200 mg/d for 17 weeks (DS).

‡ Pa 200 mg/d for 26 weeks (DR).

§ Standard of care (HRZE/HR).

BMI = body mass index; FSH = follicle stimulating hormone; Pa = pretomanid; Mfx = moxifloxacin; Z = pyrazinamide; H = isoniazid; R = rifampicin; E = ethambutol; inhB = inhibin B; LH = luteinizing hormone; T = (total) testosterone; DS = drug-susceptible; DR = drug-resistant.

**Table 5:** Summary of serum FSH, InhB, LH, T and BMI in NC-008

		Baseline		EOT		Week 39 follow-up	
		
		*n*	Median	*n*	Median	*n*	Median
FSH	BdqPaMfxZ, Week 17^*^	112	5.1	30	8.1	85	4.7
	BdqPaMfxZ, Week 26^†^	93	6.2	62	7.6	72	5.4
	HRZE, Week 26^‡^	117	5.3	58	5.0	95	4.1
InhB	BdqPaMfxZ, Week 17^*^	112	97.8	29	104.0	67	127.0
	BdqPaMfxZ, Week 26^†^	88	111.3	54	114.0	57	133.0
	HRZE, Week 26^‡^	118	101.0	51	147.0	72	139.0
LH	BdqPaMfxZ, Week 17^*^	112	7.1	30	6.5	85	5.2
	BdqPaMfxZ, Week 26^†^	93	6.7	62	6.2	72	5.7
	HRZE, Week 26^‡^	117	6.8	58	6.2	95	5.9
T	BdqPaMfxZ, Week 17^*^	112	15.4	30	20.7	85	20.9
	BdqPaMfxZ, Week 26^†^	93	18.0	62	22.1	72	25.2
	HRZE, Week 26^‡^	117	16.3	58	33.2	95	22.2
BMI	BdqPaMfxZ, Week 17^*^	110	18.9	33	19.6	84	20.0
	BdqPaMfxZ, Week 26^†^	93	18.6	62	20.1	71	20.4
	HRZE, Week 26^‡^	118	18.6	61	20.5	94	20.3

* Group treated with Pa 200 mg/d (BdqPaMfxZ, Week 17; drug-susceptible).

^†^ Group treated with Pa 200 mg/d (BdqPaMfxZ, Week 17; drug-resistant).

^‡^ Group treated with standard of care (HRZE, drug-susceptible).

FSH=follicle stimulating hormone; InhB=Inhibin B; LH=luteinizing hormone; T=testosterone (total); BMI=body mass index; EOT = end-of-treatment; Bdq = bedaquiline; Pa = pretomanid; MFX = moxifloxacin; Z = pyrazinamide; H = isoniazid; R = rifampin; E = ethambutol.

Additionally, each hormone in each of studies NC-002, NC-006, and NC-008 was evaluated separately using repeated measures analysis (Supplementary Table S2). All analyses were exploratory; there were no adjustments for multiplicity. The main objectives were to characterize changes from baseline to EOT and FU and, for NC-002 and NC-006, to compare the Pa doses of 100 mg and 200 mg.

## RESULTS

### Clinical Trial NC-002

This trial accrued ~30 male patients per group, and assessed FSH, LH and T at baseline and at 8 weeks (EOT). Pa was administered at 100 mg/d (in drug-susceptible [DS] patients) and 200 mg/d (in drug-susceptible and drug-resistant patients combined) in PaMfxZ regimen. The hormone and BMI data are summarized in Table 2. Median T levels at baseline (12.0–14.7 nmol/L) were near the 25^th^ percentile (13.7 nmol/L) of the reference range. Following 8 weeks of treatment, small FSH changes varied across groups, while LH decreased slightly. In the Pa groups, the median T levels increased by EOT to near the median level of the reference range; whereas, in the HRZE group, the median T level at EOT increased above the upper limit of the normal range. Median BMI was at or below the underweight threshold at baseline and increased into the normal weight range by EOT in all groups. Results from repeated-measures modeling are given in Supplementary Table S3 and Supplementary Figure S1. With wide, overlapping confidence intervals, percentage changes from baseline did not differ significantly between the two Pa arms.

### Clinical Trial NC-005

This trial accrued ~40 male patients per group and assessed FSH at baseline and after 4 (Day 29) and 8 weeks (Day 57) of treatment, with FU at 10 weeks (Day 70). Pa was administered at 200 mg/d in two BdqPaZ arms in DS patients (differing in the BDQ regimen) and a BdqPaMfxZ arm in DR patients (Supplementary Table S1). Results are shown in Table 3. There were no consistent changes in FSH. Median BMI values were below or slightly above underweight at baseline, and increased into or near the normal weight range by EOT and FU.

### Clinical Trial NC-006

This trial accrued ~50 male patients per group and assessed FSH, LH, InhB, and T at baseline (Week 0) and Weeks 1, 2, 4, 8, 12, 17, 26. Pa was administered at 100 mg/d and 200 mg/d in PaMfxZ for 17 weeks (DS patients), and at 200 mg/d in PaMfxZ for 26 weeks (DR patients). Results are shown in Table 4.

Median T levels at baseline (9.5–11.5 nmol/L) were near the 5^th^ percentile of the reference range (9.5 nmol/L). Across all groups, there was a progressive correction of this hypogonadism with treatment. The increase in T levels was more rapid and attained a higher level in the HRZE group. Median LH levels were within the normal reference range and did not change or fell slightly with treatment in all groups. Median FSH levels were within the normal reference range, and these levels increased rapidly with treatment and then reverted to near baseline levels by EOT or FU. The initial increase in FSH occurred in all groups, but was most pronounced in the patients treated with 200 mg/d Pa. Median InhB levels were within the normal range, and these levels increased with treatment in all groups.

Inferential analysis using repeated-measures modeling for all subjects in the two 17-week Pa treatment arms revealed a significant effect (*P* = 0.001) of Pa dose on the FSH change from baseline and no significant differences (*P* < 0.05) in the other three hormones measured. The difference in FSH levels between Pa100MfxZ and Pa200MfxZ was most pronounced at the early times points after beginning treatment; at EOT and FU, confidence intervals for percentage change overlapped (Figure 1 and Supplementary Table S4).

Median BMI levels were around the underweight threshold at baseline and increased to a similar extent in all groups into the normal weight range at EOT and recovery (Table 4).

### Clinical Trial NC-008

This trial recruited ~100 male patients into each of three groups: 1) patients with DS-TB who received BdqPaMfxZ for 4 months; 2) patients with DR-TB who received BdqPaMfxZ for 6 months; and 3) patients with DS-TB who received HRZE for 6 months. Pa was administered at 200 mg/d. FSH, InhB, LH, T, and BMI were assessed at baseline (calculated as the average of the screening and Day 1 values), EOT (Week 17 for Group 1 and Week 26 for Groups 2 and 3), and FU (Week 39). Results are shown in Table 5 and Supplementary Table S6.

Median T levels at baseline were in the middle of the normal reference range. These levels increased in all groups by EOT and FU. The HRZE group had a marked increase in T at EOT, above the upper limit of the normal reference range, which then decreased to similar levels as with the other arms at Week 39 FU. Median LH levels were within the normal reference range and fell slightly with treatment in all groups. Median FSH levels at baseline were within the normal reference range, and these levels increased by EOT in both BdqPaMfxZ groups (17 and 26 week treatments) compared to the HRZE group at EOT. By Week 39, all groups had median FSH levels below the baseline. Median InhB levels were within the normal range, and these levels increased with treatment in all groups. The median InhB in the HRZE group was more elevated at EOT than the Pa-treated groups. By Week 39, all the groups had a similar median InhB level.

Median BMI values were slightly above the underweight threshold at baseline and increased to a similar extent in all groups by EOT and recovery (Table 5). Human serum albumin levels, also reflective of overall wellbeing, increased to a similar extent in all groups between baseline and EOT (Supplementary Table S7).

The repeated-measures model included baseline BMI as a covariate and identified significant associations of baseline BMI with levels of LH (*P* = 0.01) and FSH (*P* = 0.0499). For both LH and FSH, an approximate decline of 2% in hormone level was associated with each unit decrease in BMI.

Associations between changes in BMI and changes in hormone levels are shown graphically in Supplementary Figure S2. At EOT, positive associations were apparent for InhB and T, and negative associations for FSH and LH. By FU, these associations were attenuated, although still positive for T.

## DISCUSSION

Pa, a member of the nitroimidazole class of therapeutics, is a newly FDA-approved drug for the treatment of TB. Like others of this chemical class, Pa caused testicular toxicity in rodents, but not in monkeys, in preclinical studies. Because of this observation, HPG axis hormones were monitored in males in four clinical trials.

It is common for patients with TB not to be diagnosed and treated until months after developing active infection;^23^ the patients enrolled in these clinical trials had signs and symptoms of chronic infection, including low BMI and low serum albumin levels. This is important because, at baseline, the TB-infected patients were borderline hypogonadal, with median T levels at low percentiles of a reference range, in two of the three trials where T was measured. At presentation, patients had features of both hypogonadotropic (secondary) hypogonadism and primary hypogonadism, as previously reported in patients with TB infection, debilitating chronic diseases, and significant weight loss.^4^–^8^ The improvement in BMI with treatment in all groups in all of the clinical trials, as well as the improvement in serum albumin with treatment in all groups in NC-008 supports the debilitated states of these patients with TB infection at presentation.

Hypogonadotropic hypogonadism is defined as hypogonadism resulting from hypothalamic/pituitary causes, while primary hypogonadism is defined as hypogonadism resulting from a reduced ability of the testis itself to produce T. Evidence supporting both hypogonadotropic hypogonadism and primary hypogonadism in these patients at presentation came from the longitudinal hormonal sampling in NC-006 in which both FSH and T increased in all treatment groups within 1 week of beginning treatment (Table 4 and Figure 1), indicating a treatment-related re-alignment of both HPG axis signaling and gonadal function associated with improving health. The repeated-measures modeling in NC-008 used baseline BMI as a co-variate and showed a significant association between low BMI and lower FSH and LH indicative of hypogonadotropic hypogonadism. In all four studies, BMI increased on all treatments, with median values generally starting around the underweight threshold value of 18.5 kg/m^2^ at baseline. In a graphical analysis of NC-008, which had the largest sample sizes, post-baseline increases in BMI (Supplementary Figure S2) were associated with trends of increasing InhB and T and with decreases in FSH and LH at EOT. These changes indicate amelioration of the baseline hypogonadism. In summary, by EOT, all patient groups in all the clinical trials showed resolution of their hypogonadism, accompanied by increased serum albumin levels and increased BMI.

**Figure:**
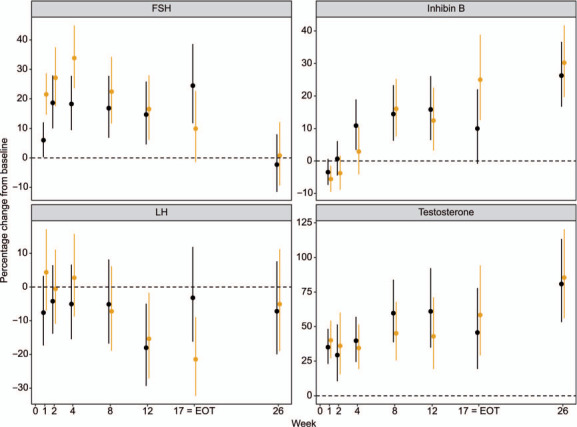
NC-006: percentage change in hormones from baseline comparing Pa_100_MfxZ-17 weeks (drug-susceptible TB) vs. PA_200_MfxZ-17 weeks (drug-susceptible TB). Dots are point estimates and vertical lines span 95% confidence intervals. Black lines = Pa_100_MZ; gold lines = Pa_200_MZ; FSH = follicle stimulating hormone; LH = luteinizing hormone; EOT =end-of-treatment.

If Pa were a testicular toxicant in humans, male reproductive tract hormones in Pa-treated patients would be expected to change to reflect damage to the testis. Such toxicity-related changes would include a decline in InhB levels, a hormone produced by the Sertoli cells, the sustentacular cells of the seminiferous epithelium, and an associated compensatory increase in FSH levels. These toxicity-related hormone changes would be expected to be associated with Pa treatment and not observed with HRZE, the standard-of-care treatment. This pattern of response was not observed in four clinical trials.

In two of the clinical trials, NC-002 and NC-006, there was a direct comparison of 100 vs. 200 mg/d Pa treatments, allowing an assessment of whether the higher Pa dose negatively affected male reproductive hormones compared to the lower dose. According to population pharmacokinetic modeling,^24^ Pa dosing follows dose proportionality under fed conditions with AUC_0–24h,ss_ (the area under the concentration-time curve from 0 to 24 hours post dose at steady state) for the 100 vs. 200 mg/d treatments of 28.8 and 57.6 μg*h/mL, respectively. For NC-002, the changes between baseline and EOT for the three hormones measured (FSH, LH, and T) were the same for the two doses of Pa (Table 2). For NC-006, when the changes between baseline and EOT (Week 17) and FU (Week 26) in DS arms for all four hormones were examined, only LH showed a significant difference between Pa doses, with the 200 mg/d group having a median LH level at EOT modestly decreased from baseline, while the Pa 100 mg/d group had a median LH level at EOT unchanged from baseline. Performing repeated-measures modeling of the longitudinal sampling in NC-006 comparing the Pa dosing arms, only FSH exhibited a significant difference between the two Pa dose groups in DS patients (Figure 1; *P* = 0.001) with the 200 mg/d Pa dose inducing a more rapid rise in FSH at early time points (Weeks 1, 2, 4, and 8). The mechanism of this significantly more rapid rise in FSH in the Pa 200 mg/d group is unknown, although one possibility is a more rapid resolution of TB infection and a more rapid reversal of the initial hypogonadotropic hypogonadism. In any case, the more rapid rise in FSH with the higher dose of Pa treatment was transient, with both the 100 and 200 mg/d doses of Pa resulting in similar hormone levels at EOT and FU.

In NC-008, median FSH levels were higher at EOT in the Pa-treated groups compared to HRZE (Table 5). Repeated-measures modeling showed that the percentage change between baseline and EOT for FSH in the HRZE group fell within the 95% confidence intervals for the Pa-treated groups (Supplementary Table S6). In addition, the FSH levels at FU for each group were lower than baseline (Table 5) and FSH percentage change from baseline to FU for all groups was similar (Supplementary Table S6).

An interesting anomaly in the hormone data was a more marked increase in serum T levels at EOT in HRZE-treated patients compared to those treated with Pa in all three studies that measured serum T (NC-002, NC-006, and NC-008). In both NC-002 and NC-008, the median serum T level for the HRZE-treated group was above the upper limit of the normal range at EOT. The pituitary hormone LH is in a negative feedback signaling loop with T; serum LH values in the HRZE-treated groups were similar to those of Pa-treated patients, with a tendency to decline slightly during treatment. The hormone measurements obtained in the NC-008 study at FU demonstrated that the elevated HRZE serum T levels at EOT were transitory and returned to the normal range after a 3-month post-exposure recovery.

A likely explanation for the HRZE-induced anomalous elevation in serum T is the known association of RIF treatment with induction of sex hormone binding globulin (SHBG).^25^,^26^ SHBG is the major serum high affinity binding protein for T.^27^ Therefore, RIF induction of SHBG would lead to an increase in plasma total T (protein-bound plus free T, the T assay performed in these clinical trials) without an increase in unbound free T, explaining the lack of effect on LH levels. It is important to note the value of having the post-exposure FU sample in NC-008 in being able to interpret the transitory nature of this anomalous HRZE-induced T elevation, and other transitory effects such as the relative increase in FSH at EOT in Pa-treated groups compared to HRZE in NC-008, which resolved by the FU time point.

Based on hormone evaluations in four clinical trials, Pa was not associated with testicular toxicity in men at the doses and exposure times evaluated. A clinical trial is ongoing to examine changes in sperm counts in men treated with Pa for active pulmonary TB (ClinicalTrials.gov Identifier: NCT04179500).
